# Long-term health benefits of physical activity – a systematic review of longitudinal studies

**DOI:** 10.1186/1471-2458-13-813

**Published:** 2013-09-08

**Authors:** Miriam Reiner, Christina Niermann, Darko Jekauc, Alexander Woll

**Affiliations:** 1 Institute of Sport and Sport Science, Karlsruhe Institute of Technology, Engler-Bunte Ring 15, 76131 Karlsruhe, Germany; 2 Institute of Sport Science. University of Konstanz, Universitätsstr. 10, 78467 Konstanz, Germany

**Keywords:** Physical activity, Adults, Weight gain, CHD, Type 2 diabetes mellitus, Dementia, NCD

## Abstract

**Background:**

The treatment of noncommunicable diseases (NCD), like coronary heart disease or type 2 diabetes mellitus, causes rising costs for the health system. Physical activity is supposed to reduce the risk for these diseases. Results of cross-sectional studies showed that physical activity is associated with better health, and that physical activity could prevent the development of these diseases. The purpose of this review is to summarize existing evidence for the long-term (>5 years) relationship between physical activity and weight gain, obesity, coronary heart disease, type 2 diabetes mellitus, Alzheimer’s disease and dementia.

**Methods:**

Fifteen longitudinal studies with at least 5-year follow up times and a total of 288,724 subjects (>500 participants in each study), aged between 18 and 85 years, were identified using digital databases. Only studies published in English, about healthy adults at baseline, intentional physical activity and the listed NCDs were included.

**Results:**

The results of these studies show that physical activity appears to have a positive long-term influence on all selected diseases.

**Conclusions:**

This review revealed a paucity of long-term studies on the relationship between physical activity and the incidence of NCD.

## Background

Especially in the last century, most Western countries have experienced significant demographic changes with a continuing increase in the number of older people who face medical and functional challenges, as well as diseases that are age-specific but have often originated in people’s younger years [1-4]. Most of these diseases including obesity, cardiovascular heart diseases (CHD) or type 2 diabetes mellitus are caused by civilisation [1,2,5]. The World Health Organisation has identified these three diseases as the most severe *noncommunicable diseases* (NCD) causing problems in today’s Western world [6]. Noncommunicable diseases are mostly diseases of slow progression and normally of long duration. The WHO identified for main types of NCDs: cardiovascular diseases, cancer, chronic respiratory diseases and diabetes [7].

Most NCDs primarily result from unhealthy lifestyles including the consumption of too much or unhealthy food [1,6,8,9], too much alcohol [1,8,10] and excessive smoking habits [1,8,11], combined with physical inactivity [1,2,8,12]. More specifically, inactivity and unhealthy eating habits are associated with weight gain, overweight and obesity are the major underlying causes for modern diseases such as CHD or type 2 diabetes mellitus [13-15]. Many cross-sectional and intervention studies have focused on the relationship between an unhealthy lifestyle, e.g. physical inactivity, unhealthy eating behaviour, smoking and alcohol consumption, and diseases in different study groups, e.g. high risk groups or different age groups [14]. All in all, cross-sectional studies suggest that physical activity may be an important factor for improving the general health and preventing the development of among others the above mentioned NCDs [1]. Because NCDs develop, not only by definition, over a long period of time and may have many causes, understanding the development of these diseases and their association with habitual factors such as physical activity is important for developing long-term prevention programs and guidelines. To investigate the development of these diseases, longitudinal studies with healthy persons, i.e. persons without obvious diseases at baseline examination, and a long term epidemiological view are necessary. It is important to follow the general population and not specific subgroups, e.g. high risk groups, persons with indications of NCD (e.g. hypertension or obesity / high body weight) or top athletes, to discover the general progression of the researched complaints in the general population.

Although these diseases are very prominent in many western countries, only few longitudinal studies exist that focus on their development during a person’s lifetime and their association with other habitual factors such as physical activity.

Many cross sectional studies have researched the relationship between physical activity and health outcomes - these results are summarized in quite a number of reviews. As opposed to this, only few long-term studies about the effect of physical activity on diseases exist, and to date there are no reviews that concentrate on long-term results in an epidemiologic view.

Therefore, the purpose of this article was to review long-term effects of physical activity on the development of weight gain and obesity, CHD and type 2 diabetes mellitus in healthy adults.

Furthermore, dementia and Alzheimer’s disease, two diseases which are of rising importance in modern societies and which develop over a long period of time, are regarded in the context of the long-term influences of physical activity. There is some evidence which indicates that physical activity has a positive effect against the development and progress of these two diseases.

## Methods

**Figure 1:**
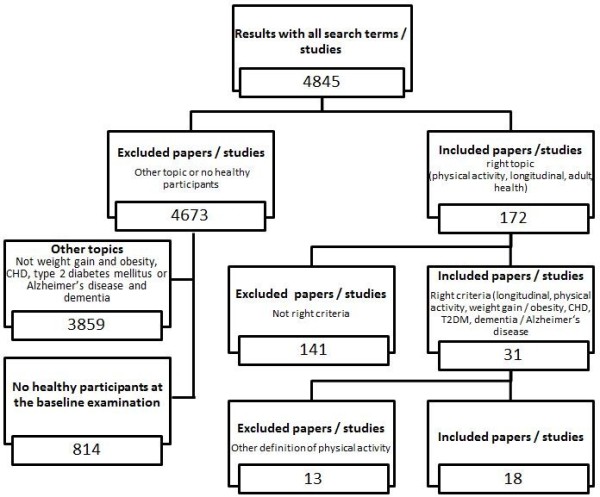
Selection criteria and number of excluded and included papers/studies.

To determine the importance of physical activity for the above described common health problems [8], only studies investigating the effect of physical activity on *weight gain* and *obesity*, *CHD*, *type 2 diabetes mellitus* and *dementia* and *Alzheimer’s disease* were included in this review. We searched the electronic databases Pubmed, BASE and OVID for articles published between January 1980 and May 2012 using the following search terms (without “and” or “or” and with longitudinal as well as long-term as a keyword to reduce the selection to such studies alone): “longitudinal / long-term, physical activity, adult” (3708 articles); “longitudinal / long-term, physical activity, adult, weight gain” (180 articles); “longitudinal / long-term, physical activity, adult, obesity” (483 articles); “longitudinal / long-term, physical activity, adult, CHD / coronary heart disease” (224 articles); “longitudinal / long-term, physical activity, adult, t2dm / type 2 diabetes mellitus” (87 articles); “longitudinal / long-term, physical activity, adult, dementia” (103 articles); and “longitudinal / long-term, physical activity, adult, Alzheimer’s disease” (60 articles) (Figure 1 Selection criteria and number of excluded and included papers / studies.).

From these studies, only *longitudinal studies with five or more years of follow-up time* were included to show the intermediate to long-term effects of physical activity rather than short-term effects of physical activity. In addition, only studies involving adults were included to show the disease development in adulthood and old age. To show the development in the general population, not in subgroups, only large epidemiological studies with *more than 500 participants* were included.

Further, only epidemiologic longitudinal studies involving *healthy adult participants* at the baseline examination were included to determine the impact of normal daily activities performed by the general population. Clinical trials, cross-sectional studies, studies involving patients, and reviews and overviews were excluded. Publications using the same study population were included as long as they held more information or investigated other topics as well.

Only those studies were included that referred to *intentional physical activity*, e.g. playing soccer, or intentional activities of daily living, e.g. take the bike for shopping, to determine the impact of leisure time physical activity in the general population. Instead of this, activities of daily living, that are necessary to live a normal self-determined life, e.g. getting up from a chair or climbing stairs, are excluded.

Finally only studies published in English were included in this review.

## Results

Overall, 4,845 articles were identified with our search strategy; of these, 4,827 were excluded from the review (Figure 1) because of the above mentioned reasons. A total of 292,278 subjects were involved at baseline (268,885 subjects at follow-up). Four publications, involving 17,329 subjects, studied the effect of physical activity on weight gain and obesity [16-19]. Six publications, involving 134,188 subjects, investigated the effect of physical activity on CHD [20-26]. Five publications, involving 84,647 subjects, studied the effect of physical activity on type 2 diabetes mellitus [27-31]. Six publications, involving 15,006 subjects, investigated the effect of physical activity on Alzheimer’s disease and dementia [32-37]. Some studies included more than one disease accounting for the discrepancy in the overall number of subjects and included studies. The maximum follow-up time ranged from 6 to 60 years.

### Effect of physical activity on weight gain and obesity

Overall, the studies included in this review showed a negative relationship between physical activity and weight gain or obesity over time. Additional file 1: Table S1 summarises the examination data and the used survey sizes for the included studies on the long-term relationship between physical activity and weight gain and obesity.

An important study analysing the development of obesity depending on physical activity is the Aerobics Center Longitudinal Study (ACLS) conducted by the Cooper Clinic, Texas [16]. Between 1970 and 1998, DiPietro et al. [16] examined 2,501 healthy men aged between 22 and 55 years at baseline and five years later. The daily physical activity level was negatively related to the weight gain during the follow-up time. Those people who reduced their daily physical activity level gained a considerable amount of weight, while those people who maintained the same level of activity during the study did not gain weight. Further, those people who increased their physical activity level during the study experienced weight loss. DiPietro et al. [16] reported that a daily physical activity level with a metabolic rate of at least 60% above the resting metabolic rate is necessary for losing weight. Hence 45 to 60 minutes of brisk walking, gardening or cycling should be included in the daily routine to maintain weight in middle-aged men.

Gordon-Larsen et al. [17] investigated the relationship between walking and weight gain. In the Coronary Artery Risk Development in Young Adults (CARDIA) Study, they examined 4,995 women and men aged between 18 and 30 years at baseline (1985/1986) who were re-examined 2, 5, 7, 10 and 15 years later. After 15 years, there was a negative association between 30 minutes walking per day and weight gain depending on the percentile of baseline weight. Data for people in the 25^th^ percentile of baseline weight showed no significant relation between walking duration and weight gain. In contrast, data for people in the 50^th^ percentile of baseline weight revealed that for every 30 minutes of daily walking the weight gain was 0.15 kg per year less for men and 0.29 kg per year less for women. Finally, data for people in the 75^th^ percentile of baseline weight showed the smallest weight gain: for every 30 minutes of walking per day, men reduced their weight gain by about 0.25 kg per year and women by about 0.53 kg per year without making any other changes to their habitual lifestyle. Hence, the results of this study indicate that participants with a higher baseline weight benefit more from being physically active (for instance, for women: the total weight gain in 15 years was 13 kg for inactive women compared to only 5 kg for active women).

Hankinson et al. [18] used the same study population (CARDIA) to investigate the physical activity level in relation to a 20-year weight gain. Of 1,561 men and women, those with high habitual activity at the 20-year follow-up had a smaller increase in mean BMI, waist circumference and weight per year compared than those with low habitual activity. Men and women maintaining higher activity gained 2.6 and 6.1 kg less weight over the 20-year period than men and women with low activity, respectively. In addition, the results of that study indicated that women benefit more from maintaining a higher physical activity level than men and that maintaining higher activity levels during adulthood may lessen weight gain during the course of their life.

The Copenhagen City Heart Study by Petersen, Schnohr and Sorensen [19] linked cross-sectional and 10 year long-term analyses, to determine the development of weight gain. They examined 3,653 women and 2,626 men at three measurement points at 5-year intervals. The participants were aged between 20 and 78 years at baseline. Results of the three cross-sectional examinations (1^st^ at baseline, 2^nd^ after five years, 3^rd^ after 10 years) also showed a negative relationship between physical activity and weight. The preventing effects of medium leisure time physical activity (LTPA) on obesity were lower than those of high LTPA for both genders. The longitudinal analysis revealed a significant direct correlation between the level of LTPA and the risk of becoming obese for men but not for women. In contrast to the results of the cross-sectional analysis, the more active participants had a higher risk of becoming obese. Moreover, the results of that study indicate that obesity may lead to physical inactivity.

Therefore, the results of the first three studies [16-18] suggest a negative correlation between physical activity and weight gain after several years of follow-up (greater physical activity leads to less weight gain). In contrast, the fourth study [19] provided evidence that being more physically active leads to a greater risk of becoming obese. They suggest that obesity influences the development of physical inactivity; however they did not discuss possible causes and effect relations. These results raise the question of the causality of the relationship between physical activity and weight gain. Detailed information, results and limitations of each study are presented in Additional file 1: Table S1.

### Effect of physical activity on coronary heart disease (CHD)

Of all modern diseases, coronary heart disease (CHD) has received the most scientific scrutiny. Overall, most studies reported a negative relationship between physical activity and the occurrence of CHD for physical activity levels above the minimum energy expenditure. Additional file 2: Table S2 summarises the examination data and the used survey sizes of the included studies addressing the longitudinal relationship between physical activity and coronary heart diseases.

In 1948, the National Heart, Lung and Blood Institute founded by Kannel et al. established the Framingham Heart Study. This research group investigated the general causes and the development of coronary heart disease in 5,209 men and women, aged 30 to 62 years at baseline [38]. The results revealed a negative association between the physical activity level and the emergence of CHD events and overall cardiovascular mortality [38-40]. Lee and Paffenbarger [20] compared the results of the Framingham Heart Study with data for 18,835 men who graduated from Harvard University between 1916 and 1950 and established the Harvard Alumni Health Study. In five mail-back surveys, researchers investigated the association between physical activity and stroke [20] and other CHD [21].

The relationship between energy expenditure and the incidence of stroke showed a u-shape pattern [20]. Specifically, spending at least 2,000 to 3,000 kcal additional energy per week on physical activity was necessary for reducing the risk of stroke. These results were reassessed for all CHD [21] in 12,516 Harvard Alumni over the course of 16 years (from 1977 through 1996). For CHD in general, the relationship between energy expenditure and the incidence of CHD showed the same u-shape pattern but the curve was shifted towards lower additional energy expenditure: spending at least 1,000 kcal additional energy per week on physical activity was necessary to reduce the risk of CHD. Hence, moderate to vigorous additional physical activity of about 2,000 to 3,000 kcal (min. 1,000 to 2,000 kcal) per week appear to reduce the overall risk for CHD, stroke and other diseases (e.g. hypertension).

Comparable results were also reported by the Honolulu Heart Program [22,23] including 8,006 men of Japanese ancestry aged 45 to 68 years at baseline who lived in Oahu, Hawaii. After 16 years, the physical activity reported at baseline was negatively related to CHD events and mortality. However, it is important to note that these results were partially mediated through the effects of hypertension, diabetes mellitus, cholesterol and BMI.

The studies cited in the next section had similar results but also featured the following additional findings.

The Alameda County Health Study by Kaplan et al. [24] reported the dependency of CHD mortality on several health factors and behaviour by quantifying the relative risks of various covariates (age, sex, perceived health, mobility impairment, heart problems, high blood pressure, diabetes mellitus, shortness of breath, current smoking, low BMI and social isolation) in 6928 men and women. After including all covariates, a protective effect of LTPA is still noticeable.

Gillum et al. [25] investigated the relationship between physical activity and stroke incidence in The National Health and Nutrition Examination Study I Epidemic Follow-Up Study on 5,852 persons aged 24 to 74 years at baseline and reported comparable results as above studies [20-23]. However, while the u-shaped relationship between physical activity and the incidence of stroke was confirmed for men, for women greater physical activity was negatively linearly associated with the incidence of stroke. In addition, recreational physical activity was not associated with the incidence of stroke in African American subjects, yet a significant interaction between heart rate and the incidence of stroke was observed only for African American subjects. The authors provided limited discussion of these differing results between Caucasian and African Americans.

To investigate the link between obesity and associated diseases, Li et al. [26] quantified the relative risk of developing CHD dependent on obesity and physical activity. They followed 88,393 Nurses aged 34 to 59 in their Nurses’ Health Study from 1980 to 2000. Being overweight and obese was significantly associated with increased risk of CHD. In addition, increased levels of physical activity were related to a graded reduction in CHD risk. Further, greater absolute mass (in kg) gained during adulthood predicted a higher CHD risk. The study concluded that obesity and physical inactivity contribute independently to the development of CHD in women.

Overall, all studies included in this review section showed a predicted negative relation between physical activity and the risk of CHD over time. Two studies [20,21] showed that a minimum additional energy expenditure of 1,000 to 2,000 kcal per week is necessary to achieve health related results. Limitations of these studies comprise the inclusion of very specific and selected participants (e.g. Harvard Alumni in the Harvard Alumni Heart Study and Nurses in the Nurses’ Health Study). In addition, these results cannot be generalized for the general public because of the selected social and ethnic backgrounds of participants and unbalanced gender distributions. In addition, most studies used Caucasian subjects alone. Hence, additional research on other ethnicities is necessary to obtain generalizable results. Moreover, the summarized studies were not designed to clarify the causality of the relationship between physical activity and CHD events. Additional research on the impact of other lifestyle factors as mediators or moderators of the relationship between physical activity and CHD is necessary. Detailed information, results and limitations of each study are presented in Additional file 2: Table S2.

### Effect of physical activity on type 2 diabetes mellitus

While the incidence of type 2 diabetes mellitus in older people has increased rapidly [1], all studies reported a negative relation between physical activity and the risk of type 2 diabetes mellitus. Additional file 3: Table S3 summarizes the results of the included studies that investigated the long-term relationship between physical activity and type 2 diabetes mellitus.

In their Nurses’ Health Study involving 70,120 nurses aged 40 to 64, which has been on-going since 1976, Hu et al. [27] investigated the relationship between participants’ physical activity level and the development of the relative risks for type 2 diabetes mellitus. Physical activity was negatively related to the incidence of type 2 diabetes mellitus even after adjusting for BMI where participants with higher physical activity levels had a lower relative risk of acquiring type 2 diabetes mellitus than those who with a lower physical activity level.

Berenzen et al. [28] and Demakakos et al. [29] reported generally comparable results in 653 men and women in the Copenhagen City Heart Study and in the English Longitudinal Study of Ageing covering different age groups, respectively. In addition to the negative relation between physical activity and the incidence of type 2 diabetes mellitus, Demakakos et al. [29] showed that moderate to vigorous physical activity (performed at least once per week) is necessary to achieve a positive effect on health and to reduce risk of type 2 diabetes mellitus. Stratifying their results by age revealed that with increasing age a higher intensity per training session or even several sessions per week are required to achieve the same risk reduction.

A high body weight or obesity, often described by the relation between body weight and body height (body mass index—BMI), and socioeconomic status are strong covariates for the relationship between physical activity and the incidence of type 2 diabetes mellitus. For instance, Katzmarzyk et al. [30] analysed the association between obesity, physical activity, cardiorespiratory fitness and the incidence of type 2 diabetes mellitus in their Physical Activity Longitudinal Study involving 1,543 men and women. Obesity and physical fitness, but not physical activity, were significant predictors of the incidence of type 2 diabetes mellitus. Mozaffarin et al. [31] added lifestyle factors in their analysis of the risk of type 2 diabetes mellitus in 4,883 participants of the Cardiovascular Health Study. Low-risk lifestyle factors included physical activity above the median level, dietary score in the upper two quintiles, having never smoked, no alcohol, a body mass index below 25 kg/m^2^ and a waist circumference below 88 cm for women or below 92 cm for men. With every healthy lifestyle factor the incidence for type 2 diabetes mellitus decreased by 35%. For people scoring lowest (that is, were the healthiest) in every lifestyle factor, an 82% lower risk for type 2 diabetes mellitus was predicted compared to all other patients. In addition, it was predicted that if these associations were causal, 8 of 10 cases of type 2 diabetes mellitus could be prevented.

All studies [28-31] reported a negative relationship between physical activity and the incident risk of type 2 diabetes mellitus. However, there are other factors than physical activity that are important in the development of type 2 diabetes mellitus. For instance, the results of the Physical Activity Longitudinal Study by Katzmarzyk et al. [31] suggest that not only the presence or absence of physical activity is a determining health factor but that the level of obesity and physical fitness also has an influence on the relationship between physical activity and the state of health. However, it is difficult to confirm these conclusions because of the small number of longitudinal studies that consider physical fitness and other lifestyle factors. In addition, the precise mechanism of how physical activity acts to reduce the risk of type 2 diabetes mellitus, such as through altered insulin sensitivity or altered insulin production, is still unknown.

### Effect of physical activity on Alzheimer’s disease and dementia

The relationship between physical activity and dementia, particularly Alzheimer’s disease, is important for the general public because the incidence of dementia increases with increasing age [1]. Additional file 4: Table S4 summarizes the results of the included longitudinal studies on the relationship between physical activity and Alzheimer’s disease and dementia.

The few existing studies [32-37] found that physical activity is negatively related to the incidence of Alzheimer’s disease and dementia in healthy men and women. Physically active people are at a lower risk of developing cognitive impairment and have a higher cognitive ability score. Interestingly, activities with low intensity, such as walking, are negatively related to the incidence of dementia and Alzheimer’s disease [32]. These results indicate that regular physical activity may be an important and potent factor preventing cognitive decline and dementia in healthy older people. Most studies on Alzheimer’s disease and dementia originate in the field of Psychology. The link between physical activity and Alzheimer’s disease and dementia in healthy participants at baseline has only been reported in very few studies [32-37], further emphasizing the overall lack of studies and specifically the lack of long-term studies that include people without dementia or Alzheimer’s disease. Most studies included people who had already been diagnosed with dementia or Alzheimer’s disease to research the development of the diseases. Detailed information, results and limitations of all included studies on physical activity and Alzheimer’s disease and dementia are presented in additional file 4: Table S4.

## Discussion

The results of the reviewed studies indicate that physical activity seems to be an important factor that can have beneficial effects for the reviewed noncommunicable diseases weight gain and obesity, CHD and type 2 diabetes mellitus, the risk factors weight gain and obesity and the age-related diseases dementia and Alzheimer’s disease.

Two of the three longitudinal studies with at least 5-year follow-up focusing on the development of obesity over time showed a negative relationship between physical activity and obesity [16,17]. Surprisingly, results of one study indicated that high leisure-time physical activity increased the risk of becoming obese in the following ten years for men [19]. The reason for this remains unexplained. Overall, the results of the studies included in this review are inconclusive regarding the required minimum level of physical activity for preventing obesity. There is no evidence for the type, intensity and frequency of activities that lead to positive health results.

Several studies [20-26,38] investigated the longitudinal effect of physical activity on the development of coronary heart diseases. Overall, the results showed a positive long-term effect where people who were physically active had a lower risk of suffering from a CHD later in their life. A minimum additional 1,000 kcal energy expenditure per week spent on physical activity has been found to be necessary for preventing overall CHD [21]. However, information on the type, intensity and frequency of activities necessary for reducing the incidence of CHD are unknown.

The results of studies [28-31] examining the effect of physical activity on the risk of suffering from type 2 diabetes mellitus showed a negative relation where higher rates of physical activity were associated with a lower risk of developing a type 2 diabetes mellitus. A higher level of physical activity appears to be required, that is a higher intensity per training session or even several sessions per week are needed, for achieving health benefits [29]. Presumably, not only physical activity level but also weight and fitness status, and their association, play a role in the development of type 2 diabetes mellitus [30].

Finally, six studies [32-37] focused on the relationship between physical activity and the incidence of dementia and Alzheimer’s disease. Results of these studies emphasized the importance of regular physical activity, but no information was provided about the type, intensity and frequency of physical activity that has the greatest health benefit.

However, several problems in the reviewed studies have become apparent.

First: There are only few long-term studies on the relationship between physical activity and the incidence of NCD, which stresses the general paucity of longitudinal research in this area. More long-term studies, following the development of diseases and the impact of lifestyle, especially physical activity, are needed. Further longitudinal studies are needed that differentiate between ethnic groups, genders and groups with different social backgrounds. The results presented in this review only encompass the relationship between physical activity and the incidence of NCD in western countries and mainly for Caucasian participants. In addition, only adults were included in these studies, and hence the results cannot be generalized to other groups. Many age related diseases, such as type 2 diabetes mellitus, CHD or certain types of cancer, develop over a long time before they are diagnosed by a physician. To identify this development in detail, longitudinal studies involving healthy participants at baseline should be conducted and these participants should be followed into older age when the disease occurs. To understand this lifelong development of NCD, studies following children throughout their lifespan are desirable. To the best of our knowledge, there are only very few studies that follow children through their adolescence into their adulthood [41,42]. The realization of this approach is very difficult, so the research should be as long as possible and about different groups, e.g. different age-related cohorts.

Second: However, more research is necessary on the clinical picture and the development of NCD. Clearly, a thorough insight into these aspects is a prerequisite for the design and development of effective prevention programs. In addition, the relationship between physical activity and the development of NCD must be better understood including the role of other parameters, such as, for instance, nutrition, body composition, alcohol consumption and smoking behaviour. Indeed, results of some of the included studies [17,18,20,21,23,24,26,27], [29,31-33,36,37] suggested that other factors including eating behaviour and food intake, smoking habits and a general activity level or disease specific risk factors such as hypertension are involved in the correlations of physical activity and health outcomes. It is almost impossible to explain the impact of just one factor, e.g. just physical activity, on the development of a lifestyle related complains like CHD - other factors are always involved, e.g. genetic constitution, other diseases, for instance obesity in the relationship with type 2 diabetes mellitus, personal behaviour or individual factors, like cognitive, motivational, volitional or emotional aspects.

Third: Most studies [16-24,26,27,29,31-38] only used self-reported/estimated physical activity for measuring participant’s physical activity. However, some studies [30,43] (this study was excluded from the review because they researched just physical fitness, not intentional physical activity), showed that the correlation of physical activity and health benefits are mediated through the physical fitness level. The quality and relevance of findings could be improved by the use of an objectively assessed variable, such as the physical fitness level measured by a fitness test or the physical activity level monitored by an accelerometer to become independent of subjective estimates and social desirability [44]. Another limitation of using self-reported physical activity alone is the fact that most questionnaires only feature the actual physical activity at the time of the examination, and hence are unable to assess physical activity performed between questionnaire administrations. However, this information is critical for determining the importance of continuous physical activity in a healthy and active lifestyle and its benefits for health [45,46].

The reviewed studies have shown that physical activity could help in the prevention of non-communicable and age-related diseases. The studies have shown that it is necessary to include physical activity into prevention programs for NCD and to inform the patients and the population in general about its virtues. To achieve this, a closer cooperation between physicians, research and sport facilities is needed. Research and physical activity service providers, e.g. gyms or sports clubs, health insurances or public providers (e.g. adult education centers) have to cooperate together to improve the general health. In addition, the knowledge about the causes and the development of modern diseases in the population should be improved. Instead of treating with medicine alone, physicians should advise patients to be more physically active within their limits. Children and adolescents should generally be encouraged to maintain a healthy lifestyle throughout their lives. In addition, public health projects that are targeted at improving the general health during adulthood and older age should focus on effective disease prevention starting during childhood.

It is important to highlight the limitations of this review in order to provide a context for the results. First, the assessment is limited to published work and may be subject to publication bias. Second, the influence of several confounders, as age, the lag between baseline and follow-up, or attrition rate, could affect conclusions of this review. Third, the work contained in this review is limited to English-written journals and thus the results cannot generalize to studies conducted and published in other languages. Fourth, we included only studies with more than 500 participants. Fifth, the literature reviewed consisted of self-reported physical activity. Finally, the review is limited to the search terms and data-bases contained in our “Methods” section. Studies that have not been abstracted with these key words will be missing from our review.

## Conclusions

This review indicates the relative lack of epidemiologic longitudinal studies on the effects of physical activity in addition to non-communicable diseases. The presented studies exclusively illustrate positive results. To the best of our knowledge no other studies reporting no or negative results over time exist.

To show the longitudinal improvements of physical activity in addition to the presented non-communicable diseases of a large number of adults within normal communities, no studies with subsamples or unhealthy participants alone were considered. This review just focuses on studies with more than 500 healthy participants. Other studies [e.g. [47] following smaller samples of participants were not included in this review; however they too contribute to the long term understanding of the development of non-communicable diseases.

Overall, the results of the reviewed articles provide a general view about the longitudinal relationship between physical activity and the incidence of NCD and health problems. Physical activity seems to be a relevant factor for preventing age-related diseases; however more long-term research is necessary.

## Competing interests

The authors declare that they have no competing interests.

## Authors’ contributions

MR searched for relevant literature and wrote the manuscript. CN and DJ helped writing and drafting the manuscript. AW has given final approval of the version to be published. All authors read and appropriated the final manuscript.

## Pre-publication history

The pre-publication history for this paper can be accessed here:

http://www.biomedcentral.com/1471-2458/13/813/prepub

## Supplementary Material

Additional file 1: Table S1 Description of studies on the association between physical activity and weight gain / obesity. Description of dataset: Table S1 describes the included studies on the association between physical activity and weight gain or obesity (Author and year, Name of the study, Baseline and measuring points, Follow up time, Baseline sample and Age at baseline, Drop out, sample size in the survey, Operationalization of physical activity and the outcome variables, Results, Limitations). Click here for file

Additional file 2: Table S2 Description of studies of the association between physical activity and coronary heart diseases. Description of dataset: Table S2 describes the included studies on the association between physical activity and coronary heart diseases (Author and year, Name of the study, Baseline and measuring points, Follow up time, Baseline sample and Age at baseline, Drop out, sample size in the survey, Operationalization of physical activity and the outcome variables, Results, Limitations). Click here for file

Additional file 3: Table S3 Description of studies on the association between physical activity and type 2 diabetes mellitus. Description of dataset: Table S3 describes the included studies on the association between physical activity and type 2 diabetes mellitus (Author and year, Name of the study, Baseline and measuring points, Follow up time, Baseline sample and Age at baseline, Drop out, sample size in the survey, Operationalization of physical activity and the outcome variables, Results, Limitations). Click here for file

Additional file 4: Table S4 Description of studies on the association between physical activity and Alzheimer’s disease and dementia. Description of dataset: Table S4 describes the included studies on the association between physical activity and Alzheimer’s disease and dementia (Author and year, Name of the study, Baseline and measuring points, Follow up time, Baseline sample and Age at baseline, Drop out, sample size in the survey, Operationalization of physical activity and the outcome variables, Results, Limitations). Click here for file

## References

[B1] Dishman RK Washburn RA Heath GW Physical Activity Epidemiology 2004 Champaign, IL: Human Kinetics

[B2] Schuit J Physical activity, body composition and healthy ageing Sci & Sports 2006 21 209 213 10.1016/j.scispo.2006.06.00424032072

[B3] Raebel MA Malone DC Conner DA Xu S Porter JA Lanty FA Health services use and health care costs of obese and nonobese individuals Arch Intern Med 2004 164 2135 3140 10.1001/archinte.164.19.2135 15505127

[B4] Chan R Woo J Prevention of overweight and obesity: how effective is the current public health approach Int J Environ Res Public Health 2010 7 765 783 10.3390/ijerph7030765 20617002 PMC2872299

[B5] Booth FW Chakravarthy MV Cost and Consequences of Sedentary Living: New Battleground for an Old Enemy 2002 Washington DC: President’s Council on Physical Fitness and Sports

[B6] Chai W Nigg CR Pagano IS Motl RW Horwath C Dishman RK Associations of quality of life with physical activity, fruit and vegetable consumption, and physical inactivity in a free living, multiethnic population in Hawaii: a longitudinal study The international journal of behavioral nutrition and physical activity 2010 7 83 10.1186/1479-5868-7-83 21092223 PMC2996342

[B7] World Health Organization Global status report on noncommunicable diseases 2010. World Health Organisation 2011 Geneva, Switzerland: WHO Press

[B8] World Health Organization Global Health Risks - Mortality and burden of disease attributable to selected major risks 2009 World Health Organization http://www.who.int/healthinfo/global_burden_disease/GlobalHealthRisks_report_full.pdf

[B9] Astrup A Dyerberg J Selleck M Stender S Nutrition transition and its relationship to the development of obesity and related chronic diseases Obes Rev 2008 9 48 52 10.1111/j.1467-789X.2007.00438.x 18307699

[B10] Rehm J Mathers C Popova S Thavorncharoensap M Teerawattananon Y Patra J Global burden of disease and injury and economic cost attributable to alcohol use and alcohol-use disorders Lancet 2009 373 2223 2233 10.1016/S0140-6736(09)60746-7 19560604

[B11] Ambrose JA Barua RS The pathophysiology of cigarette smoking and cardiovascular disease: an update J Am Coll Cardiol 2004 43 1731 1737 10.1016/j.jacc.2003.12.047 15145091

[B12] Bijnen FCH Caspersen CJ Mosterd WL Physical inactivity as a risk factor for coronary heart disease: a WHO and international society and federation of cardiology position statement Bull World Health Organ 1994 72 1 4 8131243 PMC2486506

[B13] de Berrington Gonzaley A Hartge P Cerhan JR Body mass index and mortality among 1.43 million white adults N Engl J Med 2010 363 2211 10.1056/NEJMoa1000367 21121834 PMC3066051

[B14] Vogel T Brechat P-H Leprâtre P-M Kaltenbach G Berthel M Lonsdorfer J Health benefits of physical activity in older patients: a review The Int J od Clin Pract 2009 63 303 320 10.1111/j.1742-1241.2008.01957.x 19196369

[B15] Warburton DE Nicol CW Bredin SS Health benefits of physical activity: the evidence CMAJ 2006 174 801 809 16534088 10.1503/cmaj.051351 PMC1402378

[B16] Di Pietro L Dziura J Blair SN Estimated change in physical activity level (PAL) and prediction of 5-year weight change in men: the aerobics center longitudinal study Int J Obes Relat Metab Disord 2004 28 1541 1547 10.1038/sj.ijo.0802821 15543159

[B17] Gordon-Larsen P Hou N Sidney S Sternfeld B Lewis CE Jacobs DR Popkin BM Fifteen-year longitudinal trends in walking patterns and their impact on weight change Am J Clin Nutr 2009 89 16 26 10.3945/ajcn.2008.26147 PMC2715291 19056560

[B18] Hankinson AL Daviglus ML Bouchard C Carnethon M Lewis CE Schreiner PJ Liu K Sidney S Maintaining a high physical activity level over 20 years and weight gain J Am Med Assoc 2010 304 2603 2610 10.1001/jama.2010.1843 PMC3864556 21156948

[B19] Petersen L Schnohr P Sorensen TI Longitudinal study of the long-term relation between physical activity and obesity in adults Int J Obes Relat Metab Disord 2004 28 105 112 10.1038/sj.ijo.0802548 14647181

[B20] Lee I-M Paffenbarger RS Physical activity and stroke incidence - the Harvard alumni health study Stroke 1998 29 2049 2054 10.1161/01.STR.29.10.2049 9756580

[B21] Sesso H Paffenbarger RS Lee I-M Physical activity and coronary heart disease in men - the Harvard alumni health study Circulation 2000 102 975 980 10.1161/01.CIR.102.9.975 10961960

[B22] Donahue R Abbott R Reed D Katshuiko Y Physical activity and coronary heart diseases in middle-aged and elderly men: the Honolulu heart program J Public Health 1988 78 683 685 10.2105/AJPH.78.6.683 PMC1350283 3369600

[B23] Rodriguez BL Curb JD Burchfield CM Abbott RD Petrovich H Masaki K Chiu D Physical activity and 23 year incidence of coronary heart disease morbidity and mortality among middle-aged men - the Honolulu heart program Circulation 1994 89 2540 2544 10.1161/01.CIR.89.6.2540 8205662

[B24] Kaplan GA Strawbridge WJ Cohen RD Hungerford LR Natural history of leisure-time physical activity and its correlates: associations with mortality from all-causes and cardiovascular disease over 28 years Am J Epidemiol 1996 144 793 797 10.1093/oxfordjournals.aje.a009003 8857828

[B25] Gillum RF Mussolino ME Ingram DD Physical activity and stroke incidence in women and men - the NHANES I epidemic follow up study Am J Epidemiol 1996 143 860 869 10.1093/oxfordjournals.aje.a008829 8610699

[B26] Li TY Rana JS Manson JE Willett WC Stampfer MJ Colditz GA Rexrode KM Hu FB Obesity as compared with physical activity in predicting risk of coronary heart disease in women Circulation 2006 113 499 506 10.1161/CIRCULATIONAHA.105.574087 16449729 PMC3210835

[B27] Hu FB Sigal R Rich-Edwards JW Colditz GA Solomon CG Wilett WC Speizer FE MJ E Walking compared with vigorous physical activity and risk of type 2 diabetes in women - a prospecttive study J Am Med Assoc 1999 282 1433 1439 10.1001/jama.282.15.1433 10535433

[B28] Berenzen T Petersen L Pedersen O Black E Astrup A Sorensen TI Long-term effects of leisure time physical activity on risk of insulin resistance and impaired glucose tolerance, allowing for body weight history, in Danish men Diabet Med 2007 26 63 72 10.1111/j.1464-5491.2007.01991.x 17227326

[B29] Demakakos P Hamer M Stamatakis E Steptoe A Low-intensity physical activity is associated with reduced risk of incident type 2 diabetes in older adults: evidence from the English longitudinal study of ageing Diabetologia 2010 53 1877 1885 10.1007/s00125-010-1785-x 20495973

[B30] Katzmarzyk PT Craig CL Gauvin L Adiposity, physical fitness and incident diabetes: the physical activity longitudinal study Diabetologia 2007 50 538 544 10.1007/s00125-006-0554-3 17221212

[B31] Mozzafarian D Kamineni A Carnethon M Djousse L Mukamal K Siscovick D Lifestyle risk factors and new-onset diabetes mellitus in older adults Arch Intern Med 2009 169 798 807 10.1001/archinternmed.2009.21 19398692 PMC2828342

[B32] Abbott RD White LR Ross GW Masaki K Curb JD Petrovich H Walking and dementia in physically capable elderly men J Am Med Assoc 2004 292 1447 1453 10.1001/jama.292.12.1447 15383515

[B33] Larson EB Wang L Bowen JD McCormick WC Teri L Crane P Kukull W Exercise is associated with reduced risk for incident dementia among persons 65 years of age and older Ann Intern Med 2006 144 73 81 10.7326/0003-4819-144-2-200601170-00004 16418406

[B34] Rovio S Kåreholt I Helkala E-L Viitanen M Winblad B Tuomilehto J Soininen H Nissinen A Kivipelto M Leisure-time physical activity at midlife and the risk of dementia and Alzheimer’s disease The Lancet Neurology 2005 4 705 711 10.1016/S1474-4422(05)70198-8 16239176

[B35] Laurin D Verreault R Linday J MacPerson K Rockwood K Physical Activity and Risk of cognitive impairment and dementia in Elderly Persons Arch Neurol 2001 58 498 504 10.1001/archneur.58.3.498 11255456

[B36] Chang M Jonsson PV Snaedal J Bjornsson S Saczynski JS Aspelund T Eriksdottir G Jonsdottir MK Lopez OJ Harris TB The Effect of midlife physical activity on cognitive function among older adults: AGES - Reykjavik Study J Gerontol 2010 65 1369 1374 10.1093/gerona/glq152 PMC2990266 20805238

[B37] Podewils LJ Guallar E Kuller LH Fried LP Lopez OJ Carlson M Lyketsos CG Physical activity, APOE genotype and dementia risk: findings from the cardiovascular health cognition study Am J Epidemiol 2005 161 639 651 10.1093/aje/kwi092 15781953

[B38] Sherman S D’Agostino R Cobb J Does exercise reduce mortality rates in the elderly? experience from the Framingham heart study Am Heart Study 1997 128 962 972 10.1016/0002-8703(94)90596-7 7942491

[B39] Kannel W Sorley P Some health benefits of physical activity Arch Intern Med 1979 112 820 825 464698

[B40] Kannel W Belange A D’Agostino R Israel I Physical activity and physical demand on the job and risk of cardiovascular diseases and death: the Framingham study Am Heart Study 1986 112 820 825 10.1016/0002-8703(86)90480-1 3766383

[B41] Must A Jaques PF Dallai GE Bajema CJ Dietz WH Long-term morbidity and mortality of overweight adolescents — a follow-up of the Harvard growth study of 1922 to 1935 N Engl J Med 1992 327 1350 1355 10.1056/NEJM199211053271904 1406836

[B42] Juonala M Järvisalo MJ Mäki-Torkko N Kähönen M Viikari JSA Raitakari OT Risk factors identified in childhood and decreased carotid artery elasticity in adulthood - the cardiovascular risk in young Finns study Circulation 2005 112 1486 1493 10.1161/CIRCULATIONAHA.104.502161 16129802

[B43] Sui X Lamonte MJ Ladika JN Hardin JW Chase N Hooker SP Blair SN Cardiorespiratory fitness and adiposity as mortality predictors in older adults J Am Med Assoc 2007 298 2507 2516 10.1001/jama.298.21.2507 PMC2692959 18056904

[B44] Adams SA Matthews CE Ebbeling CB Moore CG Cunningham JE Fulton J Herbert JR The effect of social desirability and social approval on self-reports of physical activity Am J Epidemiol 2005 161 389 398 10.1093/aje/kwi054 15692083 PMC2958515

[B45] Klein T Becker S Is there really a reduction in physical activity in the life-span? an analysis of age-and cohort-related differences in physical activity Z Soziol 2008 37 226 245

[B46] Klaperski S Seelig H Fuchs R Physical activity as a stress buffer Z Sportpsychol 2012 19 80 90 10.1026/1612-5010/a000061

[B47] Drygas W Kostka T Jegier A Kuński H Long-term effects of different physical activity levels on coronary heart disease risk factors in middle-aged men Int J Sports Med 2000 21 235 241 10.1055/s-2000-309 10853693

