# Supplementary material for: Long-term health benefits of physical activity – a systematic review of longitudinal studies
Source: BMC Public Health. 2013 Sep 8;13:813. doi: 10.1186/1471-2458-13-813 (PMC3847225; doi:10.1186/1471-2458-13-813)
Supplement: Additional file 1: Table S1 — Description of studies on the association between physical activity and weight gain / obesity. Description of dataset: Table S1 describes the included studies on the association between physical activity and weight gain or obesity (Author and year, Name of the study, Baseline and measuring points, Follow up time, Baseline sample and Age at baseline, Drop out, sample size in the survey, Operationalization of physical activity and the outcome variables, Results, Limitations). [file 1471-2458-13-813-S1.pdf]

Table 1: Description of studies on the association between physical activity and weight gain / obesity.

Author Year	Study	Baseline – measuring points	Follow up time	Baseline sample; Age at Baseline	Drop out	Sample in Survey	Variables - PA; Outcome	Results	Limitations
DiPietro et al. 2004 [15]	Aerobics Center Longitudinal Study - ACLS	Baseline beween 1970 - 1998 (min. 4 examinations during that time)	5 years	216,356 woman-years - 735,554 man-years; 20 - 55 years		2501 men	*PA:* 10 specific exercise-related activities within previous 3 months (frequency; duration) Physical Activity Level – energy expenditures (MET) – Daily physical activity level *Outcome:* Body weight	Daily physical activity level is inversely related to weight gain	- results just for men<br>- no common baseline<br>- longitudinal research via person-years and not via overall years<br>- just a subgroup of the basic sample
Petersen et al. 2004 [18]	Copenhagen City Heart Study	Baseline 1976/1978 - 81/83 - 92/93	10 years	14 151 men and women; 20 – 78 years	1981/1983: 11085; 1992/1993: 6542 Overall: 10 498	3,653 women. 2,626 men	*PA:* Questionnaire by Saltin & Grimby *Outcome:* Body weight	Cross-sectional Analyse<br>- Odds Ratios [with 95% Confidence Interval] for weight gain in addition to LTPA level<br>Women<br>1st survey:<br>Low LTPA:1.00 (Ref.)<br>Medium LTPA: 0.70 [0.59-0.83]<br>High LTPA: 0.51 [0.40-0.64]<br>2nd survey:<br>Men<br>1st survey:<br>Low LTPA: 1.00 (Ref.)<br>Medium LTPA: 0.71 [0.58-0.85]<br>High LTPA: 0.65 [0.52-0.80]<br>2nd survey:	- high drop out rate

PA = physical activity, MET = metabolic equivalent of task; Ref. = Reference group

								Low LTPA: 1.00 (Ref.) / Low LTPA: 1.00 (Ref.)<br>Medium LTPA:0.75 [0.63-0.91] / Medium LTPA:0.87 [0.70-1.08]<br>High LTPA: 0.58 [0.47-0.72] / High LTPA: 0.76 [0.61-0.95]<br>3rd survey: / 3rd survey:<br>Low LTPA: 1.00 (Ref.) / Low LTPA: 1.00 (Ref.)<br>Medium LTPA: 0.61 [0.49-0.76] / Medium LTPA: 0.71 [0.54-0.92]<br>High LTPA: 0.36 [0.27-0.47] / High LTPA: 0.52 [0.39-0.68]<br><br>Longitudinal Analyse:<br>- odds ratios [with 95% Confidence Interval] for becoming obese between 2nd and 3rd survey<br><br>Women / Men<br>Low LTPA: 1.00 (Ref.) / Low LTPA: 1.00 (Ref.)<br>Medium LTPA:0.93[0.59-1.45] / Medium LTPA:1.35 [0.73-2.50]<br>High LTPA: 1.35 [0.83-2.18] / High LTPA: 1.93 [1.03-3.60]	
Gordon-Larsen et al. 2009 [16]	Coronary Artery Risk Development in Young Adults - CARDIA	Baseline: 1985/1986<br>Follow up points: 1987/1988 - 1990/1991 - 1992/1993 - 1995/1996 - 2000/2001 - 200572006	20 years	5115 - total of 30,690 observations; 18 – 30 years	90% - 86% - 81% - 79% - 74%	4995 men and women - 23,633 observations	*PA:* Frequency of participation in 13 activity categories (8 vigorous, 5 moderate) over 12 months; exercise units (frequency * intensity)<br>*Outcome:* Body weight measured with a calibrated balance-beam	Inverse association between 30 minutes walking / day and weight gain related to percentile of baseline weight:<br>25th percentile:<br>m: β = -0.07 kg/y; p = .4<br>w: β = -0.12 kg/y; p < .001<br>50th percentile:<br>m: β = -0.15 kg/y; p = .03<br>w: β = -0.29 kg/y; p < .001<br>75th percentile:<br>m: β = -0.25 kg/y; p < .001<br>w: β = -0.53 kg/y; p < .001<br>women's total weight gain (15 year):<br>inactive women: +13 kg<br>active women: +5 kg	- just walking was added in an energy-index - no other sports are included

PA = physical activity, MET = metabolic equivalent of task; Ref. = Reference group

Hankinson et al. 2010 [17]	Coronary Artery Risk Development in Young Adults - CARDIA	Baseline: 1985/1986 Follow up points: 1987/1988 - 1990/1991 - 1992/1993 - 1995/1996 - 2000/2001 - 2005/2006	20 years	5115 - total of 30,690 observations; 18 – 30 years	90% - 86% - 81% - 79% - 74%	3554 men and women	*PA:* CARDIA Physical Activity History questionnaire *Outcome:* Body weight, bod height, waist circumference	Higher activity level is associated with a smaller increase in mean BMI and weight per year, compared with low activity<br><br>Mean BMI change / year relative to lower activity category (less than 340 exercise units (men) and less than 192 exercise units (women) [95 CI]<br><br>Women: Lower BMI: 1.00 (Ref.); Moderate BMI: -.05 [-.16- .05]; Higher BMI: -.13 [-.19 - .07]<br>Men: Lower BMI: 1.00 (Ref.); Moderate BMI: -.06 [-.11- .00]; Higher BMI: -.05 [-.10 - .03]<br>Total less weight gain:<br>Men – high activity: 2.6 kg<br>Women – high activity: 6.1 kg

PA = physical activity, MET = metabolic equivalent of task; Ref. = Reference group
