# Supplementary material for: Long-term health benefits of physical activity – a systematic review of longitudinal studies
Source: BMC Public Health. 2013 Sep 8;13:813. doi: 10.1186/1471-2458-13-813 (PMC3847225; doi:10.1186/1471-2458-13-813)
Supplement: Additional file 2: Table S2 — Description of studies of the association between physical activity and coronary heart diseases. Description of dataset: Table S2 describes the included studies on the association between physical activity and coronary heart diseases (Author and year, Name of the study, Baseline and measuring points, Follow up time, Baseline sample and Age at baseline, Drop out, sample size in the survey, Operationalization of physical activity and the outcome variables, Results, Limitations). [file 1471-2458-13-813-S2.pdf]

Table 2: Description of studies of the association between physical activity and coronary heart diseases.

Author Year	Study	Baseline – measuring points	Follow up time	Baseline sample; Age at Baseline	Drop out	Sample in Survey	Variables PA; Outcome	Results	Limitations
Donahue et al 1988 [21]	Honolulu Heart Program	1965 / 1968 - still continuing as Honolulu - Asia Aging Study - 1977/1979	12 years	8 006 men of Japanese ancestry; 45 – 69 years		8 006 men of Japanese ancestry	*PA:* Usual 24 hour physical activity – hours spent in basal, sedentary, slight, moderate and heavy levels of activity – Frammingham physical activity Index *Outcome:* Presence of coronary heart diseases	after 12 years of follow- up results indicate that increased levels of physical activity reported at baseline were inversely related to the risk of coronary heart diseases in both age groups<br>- Relative Risk [with 95% Confidence Interval] of definite heart diseases for active vs. inactive (Ref.) men<br>Age 45-64 years = 0.69 [0.54-0.88]<br>Age 65 and older = 0.42 [0.18-0.96]	- no information about sample size / missing / lost persons<br>- results just for men<br>- physical activity only at baseline
Rodriguez et al 1994 [22]	Honolulu Heart Program	1965 / 1968 - still continuing as Honolulu - Asia Aging Study - 1988/1990	23 years	8 006 men of Japanese ancestry; 45 – 68 years	932 men	7 074 men	*PA:* Usual 24 hour physical activity – hours spent in basal, sedentary, slight, moderate and heavy levels of activity – Frammingham	impact of physical activity index on CHD is mediated through its effects on hypertension, diabetes, cholesterol and body mass index.<br>- Relative risks [with 95% Confidence Interval] for CHD mortality by level of physical activity index among men aged 45-64 years at exam 1 – lowest tertile of physical activity index=Ref.). (see nested table below)	- results just for men

	Middle Tertile	Highest Tertile
Age	1.12 [0.88-1.44]	0.74 [0.56-0.97]
Age, Smoking	1.12	0.72

PA = physical activity; CHD = coronary heart diseases; Ref. = Reference group; BMI = body mass index

							physical activity Index<br>*Outcome:*<br>Presence of coronary heart diseases	[0.88-1.44] [0.54-0.94]<br>Age, alcohol: 1.13 [0.88-1.45]; 0.74 [0.56-0.98]<br>Age, hypertension: 1.15 [0.90-1.48]; 0.77 [0.59-1.02]<br>Age, cholesterol: 1.14 [0.89-1.47]; 0.76 [0.58-1.00]<br>Age, BMI: 1.15 [0.90-1.48; 0.79 [0.60-1.04]<br>Age, diabetes: 1.17 [0.91-1.50]; 0.79 [0.60-1.04]	
Sherman et al. 1994 [37]	Framingham Heart Study	1948 - binnial follow up actual ongoing - Baseline for this study: 1973	16 years	5 209 men and women; Baseline: 30 - 62 years; in this study: 75 years and older	4924 persons (death, no answer or missing not right age)	189 women and 96 men	*PA:*<br>Frammingham physical activity Index - usual 24 hour physical activity – hours spent in basal, sedentary, slight, moderate and heavy levels of activity<br>*Outcome:*<br>Death from all causes, incidence of and mortality from cardiac or vascular diseases	women who are more active live longer<br>- Incidence Rates [with 95% Confidence Interval] for CVD Death within 10 years<br>Women / Men<br>Least active: 1.00 (Ref.) / Least active: 1.00 (Ref.)<br>Less active: 0.53 [0.17-1.16] / Less active: 0.73 [0.17-3.12]<br>Active: 0.40 [013-1.21] / Active: 1.25 [0.28-5.55]<br>Most active: 0.77 [0.30-1.97] / Most active: 0.52 [0.09-2.94]	- just participants older than 75 years
Gillum et al. 1996 [24]	The National Health and Nutrition Examination	Baseline: 1971/1974 - 1982/1984 - 1986 - 1987	16 years	7 895 men and women; 45 – 74 years	2 043 - death, missing data,	5 852 men and women	*PA:*<br>Two questions about the habitual	regular physical activity may be beneficial in preventing stroke in women as well as in men.<br>- Age adjusted risks [with 95% Confidence Interval] for stroke incidence associated with	- self-reported data of stroke without confirming hospital records

PA = physical activity; CHD = coronary heart diseases; Ref. = Reference group; BMI = body mass index

	Survey I (NHANES I) Epidemic Follow-up Study				stroke history		physical activity: “Do you get much exercise in things you do for recreation, or hardly any exercise or in between? ”You’re your usual day aside from recreation, are you physically very active, moderately active, or quite inactive?<br>*Outcome:* Incidence of stroke	recreational physical activity level (high physical activity level = Reference)<br><br>Women 45-64 years<br>Moderate activity: 1.84 [0.53-6.29]<br>Low Activity: 3.37 [1.04-10.98]<br><br>Men 45-64 years<br>Moderate activity: 1.04 [0.55-1.97]<br>Low Activity: 1.22 [0.64-2.33]<br><br>Women 65-74 years<br>Moderate activity: 1.32 [0.79-2.20]<br>Low Activity: 1.68 [1.03-2.73]<br><br>Men 65-74 years<br>Moderate activity: 0.89 [0.60-1.31]<br>Low Activity: 1.43 [0.98-2.08]	- only two questions about physical activity to classify the persons
Lee & Paffenbarger 1998 [19]	Harvard Alumni Health Study	Baseline: Mailsurvey 1977 - Mail back 1988	11 years	17 835 male graduates from Harvard University between 1916 – 1950; 43 – 88 years	6 705 men - missing data, physician diagnosed CHD or cancer	11,130 male graduates from Harvard Uni-versity between 1916 and 1950	*PA:* Estimation of daily blocks walks, flights of stairs climbed or to list all sports or recreation in which they had actively participated during the past year. – total energy expenditure, average	physical activity is associated with decreased risk of stroke in men – u-shaped association<br>- Relative risks [with 95% Confidence Interval] of stroke in addition to energy consumption in kcal per week<br><br><1.000 kcal / wk = 1.00 (Ref.)<br>1.000-1.999 kcal/wk = 0.76 [0.59-0.98]<br>2.000-2.999 kcal/wk = 0.54 [0.38-0.76]<br>3.000-3.999 kcal/wk = 0.78 [0.53-1.15]<br>>4.000 kcal/wk = 0.82 [0.58-1.14]	- special Subgroup (well-educated, mostly Caucasian)<br>- stroke were ascertained through self-report and death certificates - misclassification<br>- no dietary information available<br>- results just for men

PA = physical activity; CHD = coronary heart diseases; Ref. = Reference group; BMI = body mass index

							weekly energy expenditure, kilocalories per week from walking *Outcome:* diagnosis of stroke		
Sesso et al. 2000 [20]	Harvard Alumni Health Study	Baseline: Mailsurvey 1977 - Mail back 1988 or 1993	11 / 16 years	17 835 male graduates from Harvard University between 1916 – 1950; 39 – 88 years	5 319 men - missing data, physician diagnosed CHD or cancer	12 516 male graduates from Harvard University between 1916 and 1950	*PA:* Estimation of daily blocks walks, flights of stairs climbed or to list all sports or recreation in which they had actively participated during the past year. – total energy expenditure, average weekly energy expenditure, kilocalories per week from walking *Outcome:* Self-report of CHD	total physical activity and vigorous activities showed the strongest reductions in CHD risk<br>- Relative risks [with 95% Confidence Interval]of CHD in addition to energy expenditure in kJ per week (4.2 kJ = 1 kcal)<br><br><2100 kJ/wk = 1.00 (Ref.)<br>2100-4199 kJ/wk = 0.90 [0.79-1.03]<br>4200-8399 kJ/wk = 0.81 [0.71-0.92]<br>8400-12599 kJ/wk = 0.80 [0.69-0.93]<br>>12 600 kJ/wk = 0.81 [0.71-0.94]	- measurement of physical activity may lead to misclassification<br>- no description of activity during the follow- up period - physical activity levels are likely fluctuated<br>- results just for men<br>- special Subgroup (well-educated, mostly Caucasian)<br>- no control of dietary habits
Li et al. 2006 [25]	Nurses' Health Study	Baseline: 1979 - 1980	retrospective questions about weight	121 700 female registered nurses; 30 – 55 years	33 307 women	88 393 women	*PA:* Average number of hours spend each week	Association between activity, weight and risk of CHD<br>- Hazard Ratios [with 95% Confidence Interval] for weight gain in addition to physical activity level and baseline body weight	- Retrospective questions about weight with 18 years<br>- Results just for women / Nurses

PA = physical activity; CHD = coronary heart diseases; Ref. = Reference group; BMI = body mass index

	with 18 years	during the past year on moderate and on vigorous physical activities<br>*Outcome:* Incidence of myocardial infarction – review by physician	Sedentary – normal weight: 1.48 [1.24-1.77]<br>Active – obese: 2.48 [1.84-3.34]<br>Sedentary – obese: 3.44 [2.8-4.21]	- No assess of cardiorespiratory fitness

PA = physical activity; CHD = coronary heart diseases; Ref. = Reference group; BMI = body mass index
