# Supplementary material for: Long-term health benefits of physical activity – a systematic review of longitudinal studies
Source: BMC Public Health. 2013 Sep 8;13:813. doi: 10.1186/1471-2458-13-813 (PMC3847225; doi:10.1186/1471-2458-13-813)
Supplement: Additional file 3: Table S3 — Description of studies on the association between physical activity and type 2 diabetes mellitus. Description of dataset: Table S3 describes the included studies on the association between physical activity and type 2 diabetes mellitus (Author and year, Name of the study, Baseline and measuring points, Follow up time, Baseline sample and Age at baseline, Drop out, sample size in the survey, Operationalization of physical activity and the outcome variables, Results, Limitations). [file 1471-2458-13-813-S3.pdf]

Table 3: Description of studies on the association between physical activity and type 2 diabetes mellitus.

**Author Year**	**Study**	**Baseline – measuring points**	**Follow up time**	**Baseline sample; Age at Baseline**	**Drop out**	**Sample in Survey**	**Variables – PA; Outcome**	**Results**	**Limitations**
Hu et al 1999 [26]	Nurses' Health Study	Baseline: 1979 - 1986	7 years	121 700 female registered nurses; 30 – 55 years	51 597 women	70 103	*PA:* Average number of hours spend each week with certain activities; Asking about their usual walking speed – weekly energy index was calculated *Outcome:* Diagnosis of diabetes	The physical activity score is inversely associated with the risk of type 2 diabetes mellitus<br>- Relative Risk [with 95% Confidence Interval] for t2dm in addition to energy expenditure<br>1st Quintile = 1.00 (Ref.)<br>2nd Quintile = 0.77 [0.66–0.90]<br>3rd Quintile = 0.75 [0.65–0.88]<br>4th Quintile = 0.80 [0.69-0.93]<br>5th Quintile = 0.54 [0.45–0.64]	- Results just for women<br>- Self-reported answers about diabetes<br>- Just leisure time physical activity – no leisure time physical activity may also be important for diabetes prevention
Berenzen et al 2007 [27]	Copenhagen City Heart Study	Baseline: 1976/1978 - 1981/1983 - 1991 - 1998/2000	24 years	5 531 men; Median age 19 years	4 878 men	653 men – (299 obese and 354 control group)	*PA:* One question about physical activity habits: inactivity – moderate activity – high activity – very high activity *Outcome:* Diagnosis of diabetes – WHO standardized	Leisure time physical activity reduces the risk of insulin resistance and impaired glucose tolerance<br>- odds ratios [with 95% Confidence Interval] for impaired glucose tolerance in addition to intensity of activity<br>Active = 1.00 (Ref.)<br>Moderate Active = 1.72 [1.09–2.72]<br>Inactive = 4.13 [1.96–8.86]	- results just for men<br>- high dropout rate<br>- drop out people are less active and had lightly higher BMI<br>- insulin sensitivity only measured at follow-up<br>- BMI inaccurate measure for body composition

PA = physical activity, BMI = body mass index; WHO = World Health Organization; MET = metabolic equivalent of task

							oral glucose tolerance test		
Katzmarzyk et al 2007 [29]	Physical Activity Longitudinal Study	Baseline: 1981 or 1988 - 2002/2004	16 – 21 years	4 900 men and women; 18 - 69 years	3 357 men and women	1,543 persons - 709 men and 834 women	*PA:* Maximal Oxygen consumption - Canadian Aerobic Fitness Test *Outcome:* Self-reported physician diagnosed diabetes	Physical activity, physical fitness and obesity are related to incidence of type 2 diabetes mellitus<br>- Odds Ratios [with 95% Confidence Interval]for t2dm in addition to obesity measurements<br>BMI = 2.02 [1.63-2.50]<br>Waist Circumference = 2.56 [1.93–3.43]<br>Waist-Hip Ratio = 3.00 [2.03–4.46]<br>Skin fold thickness = 2.14 [1.61-2.85]<br>Physical activity = 0.77 [0.58–1.00]<br>Max. MET = 0.28 [0.14–0.57]<br>Physical fitness = 0.38 [0.23–0.62]	- self-reported physician-diagnosed diabetes<br>- high dropout rate
Mozaffarin et al 2009 [30]	Cardiovascular Health Study	Baseline: 1989/ 1990 - 1992/1993 - annual to 1999 – semi-annual by telephone - end 2005	16 years	5 888 men and women; 65 years and older	1 005 men and women	4,883 persons – 34,539 person-years	*PA:* Usual walking habits (average pace and distance) - modified Minnesota Leisure-Time Activities questionnaire *Outcome:* Incidence of diabetes	Lifestyle factors are associated with reduced risk of type 2 diabetes mellitus<br>- Hazard Ratios [with 95% Confidence Interval] for t2dm in addition to number of risk factors<br>2 factors = 0.54 [0.38-0.76]<br>3 factors – never smoke = 0.42 [0.25–0.71]<br>3 factors – no alcohol use = 0.32 [0.18–0.55]<br>4 factors = 0.18 [0.06–0.56]<br>5 factors = 0.11 [0.01–0.76]	- limited time points for fasting and post challenge glucose levels - misclassification of diabetes cases<br>- less lifestyle factors as mediator or moderators
Demakakos et al 2010 [28]	English Longitudinal Study of Ageing	Baseline: 1998/2001 – 2004/2005 – 2006/2007	9 years	11 523 men and women; 50 years and older	4 057 men and women	7 466 men and women	*PA:* How often taking part in three different types of physical activity – vigorous,	Increasing physical activity intensity decreases the risk of type 2 diabetes mellitus<br>- Hazard Ratios [with 95% Confidence Interval] for td2m in addition to intensity of activity<br>No physical activity = 1.00 (Ref.)<br>Low intensity = 0.68 [0.46–1.01]<br>Moderate / vigorous = 0.41 [0.28–0.59]	- Results just for people 50 years and older<br>- Use of self-reported physical activity<br>- Self-reported diabetes data

PA = physical activity, BMI = body mass index; WHO = World Health Organization; MET = metabolic equivalent of task

							moderate, low intensity<br>*Outcome:* Incidence of self-reported doctor-diagnosed diabetes	intensity		

PA = physical activity, BMI = body mass index; WHO = World Health Organization; MET = metabolic equivalent of task
