# Supplementary material for: Long-term health benefits of physical activity – a systematic review of longitudinal studies
Source: BMC Public Health. 2013 Sep 8;13:813. doi: 10.1186/1471-2458-13-813 (PMC3847225; doi:10.1186/1471-2458-13-813)
Supplement: Additional file 4: Table S4 — Description of studies on the association between physical activity and Alzheimer’s disease and dementia. Description of dataset: Table S4 describes the included studies on the association between physical activity and Alzheimer’s disease and dementia (Author and year, Name of the study, Baseline and measuring points, Follow up time, Baseline sample and Age at baseline, Drop out, sample size in the survey, Operationalization of physical activity and the outcome variables, Results, Limitations). [file 1471-2458-13-813-S4.pdf]

Table 4: Description of studies on the association between physical activity and Alzheimer's disease and dementia.

| **Author Year** | **Study** | **Baseline – measuring points** | **Follow up time** | **Baseline sample; Age at Baseline** | **Drop out** | **Sample in Survey** | **Variables – PA; Outcome** | **Results** | **Limitations** |
|---|---|---|---|---|---|---|---|---|---|
| Laurin et al. 2001 | Canadian Study of Health and Aging | Baseline: 1991/1992 – 1996/1997 | 5 years | 9 008 men and women – 6 434 cognitive normal persons; 65 years and older | 4 393 men and women | 4 615 men and women | *PA:* Older Americans Research Scale *Outcome:* Modified Mini-Mental State Examination; Neuropsychol-ogical test batters | High levels of physical activity are associated with reduced risk<br>- Relative Risks [with 95% Confidence Interval] for cognitive impairment, Alzheimer's disease and dementia<br>cognitive impairment = 0.58 [0.41-0.83]<br>Dementia = 0.63 [0.40-0.98]<br>Alzheimer's Disease = 0.50 [0.28-0.90] | - Characterisation of physical activity |

PA = physical activity; Ref. = Reference group

| | | | | | | | | | |
|---|---|---|---|---|---|---|---|---|---|
| Abbott et al. 2004 | Honolulu Heart Program | 1965 / 1968 - still continuing as Honolulu - Asia Aging Study – 1994/1996 – 1997/1999 | 29 – 34 years | 3 734 men of Japanese ancestry- survivors of the original Honolulu Heart Program cohort; 71-93 years | 1 477 men | 2 257 men | *PA:* Average amount of distance walked per day; Physical Activity Index<br>*Outcome:* Cognitive Abilities Screening Instrument; Informant Questionnaire on Cognitive Decline in the Elderly | Estimated relative risk of developing of dementia are associated with the distance walked per day<br><br>Total dementia / Alzheimer's disease<br>>2m/d: Ref. / Ref.<br><0.25 m/d: 2.12 / 2.24<br>0.25-1 m/d: 2.06 / 2.21<br>1-2 m/d: 1.5 / 1.33 | - Results just for men<br>- High age participants |
| Rovio et al 2005 | CADIE - Cardiovascular risk factors Aging and Incidence of Dementia | Baseline: 1972/1977/1982 or 1987 - 1998 | 11-26 years | 2000 men and women; 65-79 years | 551 men and women | 900 women, 549 men – 1449 participants | *PA:* Questions about the physical activity: "How often do you participate in leisure-time physical activity that lasts at least 20-30 min and causes breathlessness and sweating?"<br>*Outcome:* Incidence of dementia and Alzheimer's | Regular physical activity may reduce the risk or delay the onset of dementia and Alzheimer's disease<br>- Odds Ratios [with 95% Confident Interval] for developing dementia and Alzheimer's disease for active people<br><br>sedentary = Ref.<br>Dementia = 0.55 [0.30-1.01]<br>Alzheimer's Disease = 0.45 [0.22-0.93] | - Categorical ranking of physical activity – no accurate quantify<br>- No changes of physical activity during the follow-up time<br>- No intensity or frequencies of physical activity<br>- Different follow up times |

PA = physical activity; Ref. = Reference group

| | | | | | | | | | |
|---|---|---|---|---|---|---|---|---|---|
| | | | | | | | Disease | | |
| Podewils et al. 2005 | Cardiovascular Health Cognition Study | Baseline:<br>1992 - 2000 | 8 years | 5 888 men and women; 65 years and older | 2 228 men and women | 3 660 men and women | *PA:*<br>Modified Minnesota Leisure Time Physical Activity questionnaire<br>*Outcome:*<br>Cognitive Status Test, Cognitive Decline in the Elderly Test | Inverse association between physical activity level and incidence of dementia in later life.<br>Hazard Ratios [95%CI] of incident dementia by level of leisure-time energy expenditure<br><br>(columns: All-cause dementia / Alzheimer's disease)<br><248 kcal/wk: Ref. / Ref.<br>248-742 kcal/wk: 1.03 [0.81-1..31] / 0.99 [0.71-1.38]<br>743-1,657 kcal/wk: 0.81 [0.63-1.05] / 0.79 [0.56-1.12]<br>>1,657 kcal/wk: 0.74 [0.57-0.96] / 0.64 [0.45-0.93] | - High dropout rate<br>- Measurement of physical activity (Reliability of Minnesota Leisure Time Physical Activity Questionnaire) |
| Larson et al 2006 | Adults Changes in Thought | Baseline:<br>1994/1996 – 2000/2002 | 6 years | 6 782 men and women in study – Subgroup of 2 581 men and women; 65 years and older | 841 men and women | 1 740 men and women | *PA:*<br>Number of days a week the participants did each of presented exercises for at least 15 minutes at a time during the past year<br>*Outcome:*<br>Cognitive Ability Screening Instrument | Regular exercise reduces the risk of dementia<br>- Hazard Ratio [with 95% Confidence Interval] for developing dementia<br>regular activity: 0.62 [0.44-0.86]<br>- Incident rate for 1,000 person years<br>13.0 for >3 times active per week<br>19.0 for less than 3 times per week | - measurement of physical activity |
| Chang et al. 2010 | AGES – Reykjavik | Baseline:<br>1967 - 2002 | 26 - 35 years | 5 764 men and | 819 men and | 2 093 men, 2 | *PA:*<br>Questions | Inverse association between midlife physical activity and cognitive function in later life. | - Measurement of physical activity |

PA = physical activity; Ref. = Reference group

| | | | | | | | | | |
|---|---|---|---|---|---|---|---|---|---|
| | Study – Age, Gene / Environment Susceptibility Reykjavik Study | | | women; Born between 1907 - 1935 | women | 852 women – overall 4 945 | about the biography of physical activity during adult life and how many hours per week physical activity during winter and summer<br>*Outcome:* Digit symbol substitution test, figure comparison, modified Stroop Test California Verbal Learning Test, Digits Backward, CANTAB Spaital Working Memory test | Odds Ratios of having dementia mong physically active groups compared with none physical active group [95% CI]<br>No physical activity: 1.00 (Ref.)<br><5 h/wk: 0.59 [0.40 – 0.87]<br>>5 h/wk: 0.74 [0.34 – 1.62] | - No information about changes in physical activity |

PA = physical activity; Ref. = Reference group
